# Congenital hyperinsulinemic hypoglycemia (HH) requiring treatment as the presenting feature of Kabuki syndrome

**DOI:** 10.1002/ccr3.7336

**Published:** 2023-05-26

**Authors:** Saloua Ait Souabni, Antoine Harvengt, Camille Legat, Philippe A. Lysy

**Affiliations:** ^1^ Pediatric Endocrinology, Specialized Pediatrics Service Cliniques Universitaires Saint Luc, UCLouvain Brussels Belgium

**Keywords:** congenital hyperinsulinism, diazoxide, Kabuki syndrome, lanreotide, neonatal hypoglycemia, X‐linked Kabuki syndrome

## Abstract

Kabuki syndrome is a congenital condition characterized by a set of facial dysmorphic features that often help the clinician to suspect the diagnosis. However, more insidious symptoms can rarely occur, such as manifestations of hypoglycemia in newborns with congenital hyperinsulinism hypoglycemia, especially when a variant of the *KDM6A* gene is found. In those cases, a treatment with diazoxide can be started and can be replaced with lanreotide if a satisfying glycemic control is not achieved. We report the case of a female patient born at 37 weeks of gestational age, without any obvious facial dysmorphic features, after a non‐complicated pregnancy, that presented with feeding difficulties, drowsiness, and irritability revealing a hyperinsulinemic hypoglycemia. Further testing at 6 months old found a *KDM6A* mutation. The patient was initially treated by diazoxide alone, but its dosage had to be lowered because of the occurrence of treatment side effects, and lanreotide had been added to maintain acceptable blood sugar levels. A congenital hyperinsulinemia hypoglycemia revealed heterozygous truncating variant in the *KDM6A* gene, also known as X‐linked Kabuki syndrome in a newborn. In cases of neonatal hypoglycemia, the first‐line therapy is diazoxide. Our report shows that analogues of somatostatin such as lanreotide should be considered if the diazoxide regimen is not tolerated.

## Graphical abstract

Xxxx.

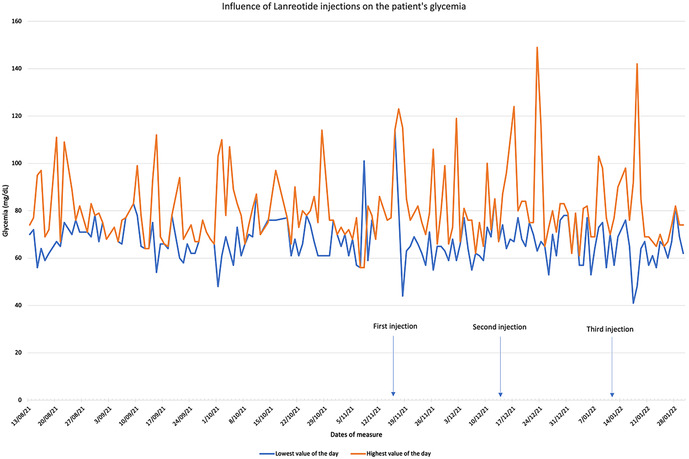

## 1 BACKGROUND

Congenital hyperinsulinism (HI) is one of the most common causes of refractory and severe hypoglycemia in the neonatal period.^1^, ^2^ Affected newborns are frequently macrosomia with a variety of symptoms such as fasting intolerance, feeding difficulties, and persistent hypoglycemia. Hypoglycemic episodes are characterized by low blood glucose level with abnormally high insulin level, absence of ketones, and no increase in plasma fatty acids. Usually, HI is mainly caused by monogenic diseases with mutations in genes involved in the regulation of insulin secretion such as *ABCC8* (11p15.1) and *KCNJ11* (11p15.1).^3^ However, HI is also a feature of more than 30 genetic syndromes as Beckwith–Wiedemann, Sotos, and Kabuki syndromes.^4^, ^5^

Kabuki syndrome is a rare, incidence of 1/32,000 births, syndrome of multisystem congenital anomalies characterized by five cardinal manifestations: dysmorphic facial features, skeletal abnormalities, growth hormone deficiency, moderate to severe intellectual deficiency, and dermatoglyphic abnormalities. Cardiac abnormalities, feeding disorders, congenital hypothyroidism, immune deficiencies, or certain neurological disorders are other possible clinical signs in KS.^6^ Neonatal hypoglycemia occurs in 10% of KS; however, HI is less frequent in patients with KS (0.3%–1%). In the etiological evaluation, a genetic mutation, often de novo is found in nearly 80% of patients. The most frequent (75% of cases) is a mutation of the *KMT2D* gene (12q13.13) followed by a variant of the *KDM6A* gene (Xp11.3).^4^ Furthermore, *KDM6A* variant (X‐linked dominant Kabuki syndrome 2, KS2) is more likely associated with HI than *KM2TD* variant (autosomal dominant Kabuki syndrome 1, KS1).^4^, ^7^

Finally, glycemic control in patients with HI is mostly achieved with diazoxide. However, some patient still present hypoglycemia under diazoxide treatment. Long‐acting lanreotide is an efficient treatment improving glycemic control.^8^, ^9^, ^10^

## 2 PATIENT PRESENTATION

### 2.1 Clinical history

We describe the case of a female newborn at 37 + 6/7 weeks of gestation after a non‐complicated pregnancy. She is the third child of two healthy, non‐consanguineous parents of Caucasian origins. Her siblings were healthy. The prenatal ultrasound tests on the first, second, and third trimesters were normal and birth weight was 3950 g, birth length 50 cm, and Apgar score 6/8/9.

On Day 1, she presented feeding difficulties as well as irritability and drowsiness revealing refractory hypoglycemia at 14 mg/dL. Hypoglycemia was eventually treated with a high glucose 10% infusion rate (>8 mg/kg/min) and continuous parenteral nutrition consisting of artificial milk enriched with maltose dextrin (7.5 mg/kg/min). A few hours later, a second episode of hypoglycemia (33 mg/dL) occurred. During this second hypoglycemia, additional blood samples were taken and showed HI with a high insulin level at 16 mUI/L. The rest of the blood test showed a satisfactory hormonal balance and an absence of ketone bodies. To treat the hypoglycemia and to avoid any recurrence, an intravenous treatment with Glucagon was first administered for 24 h followed by a continuous infusion of glucose 20% (from Day 2 to Day 6) and then glucose 10% (from Day 7 to Day 8). Further investigations (MRI, EEG, metabolic tests, abdominal ultrasound, and cardiac ultrasound) did not reveal any abnormality except a persistent oval foramen. After a recurrence of hypoglycemic episodes following the discontinuation of glucose infusion, a treatment with diazoxide at a dose of 5 mg/kg/d was started on Day 12 and progressively decreased to finally stop on Day 23 as the patient had satisfying glycemic values and digestive intolerance secondary to the medication. However, prolonged periods of fasting caused reoccurrences of hypoglycemic episodes and justified the diazoxide to be resumed on Day 29. We increased the dose progressively until we reached 15 mg/kg/d at Day 42 when satisfying serum glucose levels were achieved.

In addition to the glycemic disturbances, the patient also occurred with multiple feeding difficulties requiring nasogastric tube feeding at first, then gastrostomy (Day 62) following non‐increased PO intake. In addition, a proton pump inhibitor and domperidone, after exclusion of a long QTc by electrocardiogram, were respectively started on Day 10 and Day 35 to relieve symptoms of gastroesophageal reflux and esophageal dysmotility. Finally, further neurodevelopmental evaluation revealed that the patient also presented progressive neurodevelopmental delay.

After 73 days of hospitalization, the patient was discharged from the neonatal intensive care unit and returned home. Her last clinical examination before returning home was normal. Only morphologic clinical examination revealed moderate hypotonia, some dysmorphic features such as a moderate disproportion of the upper, middle, and lower thirds of the face, elongated eyes, without any prominent eversion of the lateral third of the lower eyelid. However, there were no ear deformities, no elongated philtrum, and no lower lip pits or other classic facial dysmorphic features.

**FIGURE 1:**
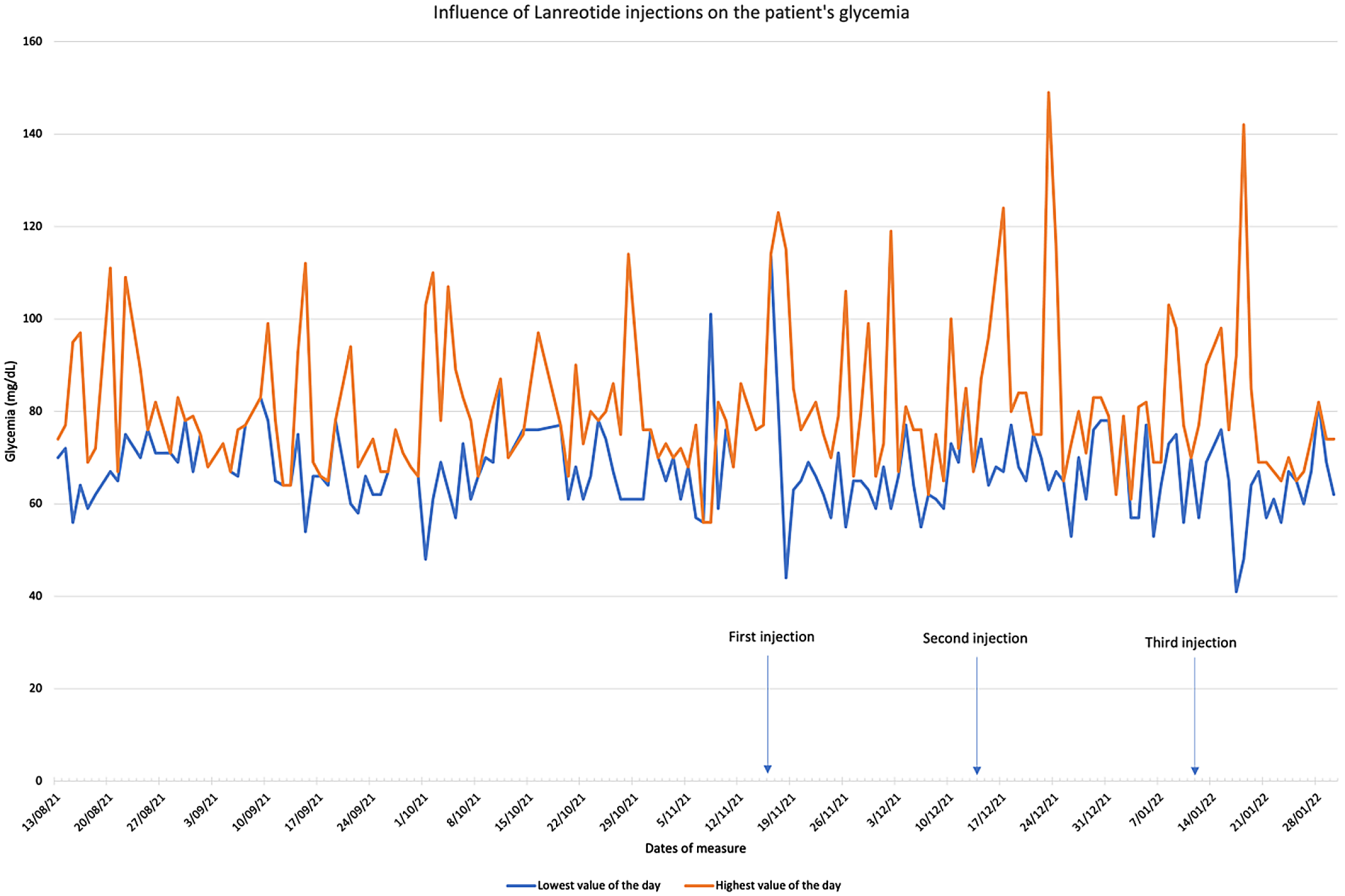
Influence of lanreotide injections on the patient's glycemia.

After her stay in neonatology, the patient was monthly seen in pediatric endocrinology consultation. While hypoglycemia was rare, diazoxide had caused feeding difficulties that warranted lowering her dosage to 10 mg/kg/d at a frequency of three times daily and prescribing somatostatin analogues to help maintain acceptable blood glucose levels. Lanreotide therapy (increased to 90 mg/month) was introduced at 7 months of age and given for a total of 3 months. The introduction of Lanreotide made it possible to reduce the doses of diazoxide and improve the infant's comfort without negatively impacting glycemic control (Table 1 and Figure 1).

**TABLE 1:** Glycemic parameters according to chronic treatment.

Glycemic parameters according to chronic treatment					
	Diazoxide 15 mg/kg/j	Diazoxide 10 mg/kg/j + lanreotide 30 mg	Diazoxide 10 mg/kg/j + lanreotide 60 mg	Diazoxide 10 mg/kg/j + lanreotide 90 mg	*p*‐value
Glycemic mean ± SD (mg/dL)	73.7 ± 7.2	75.1 ± 11.9	76.1 ± 11.2	71.3 ± 9.1	0.63^a^
Hypoglycemia frequency (*n*/day)	0.11	0.24	0.21	0.21	0.11^b^
Time spent in hypoglycemia (%)	5	12	11	11	0.32^b^

*Note*: Plus‐minus values are means ± SD. Glycemic parameters were evaluated monthly at each clinical consultation. Differences between chronic treatments were considered as significant when *p*‐value was under 0.05.

^a^ Kruskal–Wallis.

^b^ Chi‐square.

### 2.2 Genetic diagnosis

At 6 months old, the results of a genetic test that was conducted 3 months prior showed a negative hyperinsulinism panel. A next‐generation sequencing was also performed after exome enrichment using the Twist Human Core Exome kit with additional probes for human RefSeq transcripts and the mitochondrial genome (Twist Bioscience). It revealed a heterozygous truncating class 5 variant induced by a c.493C>T mutation in the KDM6A gene known as causing an X‐linked dominant KS.

## 3 DISCUSSION AND CONCLUSIONS

In this patient, the persistence of congenital HI was the presenting feature of KS. In the case of persistent HI, especially if associated with other features (feeding difficulties, neurodevelopmental delay, dysmorphic features), syndromic HI should be considered and therefore complementary genetic analyses should be performed.

Patients with pathogenic *KDM6A* variants can present atypical features of KS, such as variable facial dysmorphism, which makes a KS diagnosis difficult.^11^ Therefore, *KDM6A* sequencing for patients presenting a *KMT2D*‐mutation‐negative KS, would be justified.^12^

Congenital hyperinsulinism is one of the most common causes of severe neonatal hypoglycemia and is characterized by abnormally high insulin levels relative to glycemic values and the absence of ketone bodies or fatty acid elevation.^1^, ^2^, ^3^ HI has an incidence of approximatively 1 in every 50,000 births,^13^ with at least 11 known monogenic forms of HI and other associated syndromes including Beckwith–Wiedemann syndrome and Turner syndrome.^4^, ^5^ KS is the second most common causes of syndromic HI and 70% of children with KS have congenital HI, while 11% present neonatal hypoglycemia.^14^ However, less than 10% of neonatal hypoglycemias are caused by HI.^4^, ^15^ Other possible causes of neonatal hypoglycemia in KS include growth hormone or glucocorticoid deficiencies.^16^, ^17^ The genetic mutations responsible for HI in KS are in 70–75% of cases secondary to autosomal recessive mutations in the *KMT2D* gene and in 1–9% of cases to X‐linked mutations in *KMD6A* like in our case.^4^, ^14^ Pathogenic variants in these two genes cause abnormal chromatin remodeling, resulting in genome‐wide effects impacting a range of organ systems.^18^

A recent study showed that hypoglycemia is the most common symptom in Kabuki syndrome caused by a mutation in *KMD6A*.^17^ The *KDM6A* gene is responsible for the expression of a lysine demethylase 6A, which is a histone demethylase. A mutation of the histone‐demethylase gene, which is crucial to its normal expression, causes an inappropriate methylation of the gene histones which interacts with its correct functioning.^19^ While the *KDM6A* gene seems to be involved in epigenetic modifications during pancreatic differentiation and in insulin secretion,^20^, ^21^ the exact mechanism responsible for the insulin hypersecretion in KS is still not known.^22^

In the context of HI, a glycemic control will mostly be achieved with diazoxide, a molecule that opens the K^+^/ATP channels in the beta‐cells of the pancreatic islets, leading to hyperpolarization of beta cell and consequently and inhibition of insulin secretion.^23^ Diazoxide is the first‐line treatment in cases of neonatal hypoglycemia. Before starting treatment, a cardiac ultrasound should be performed to exclude pulmonary hypertension.^24^ Finally, digestive disorders, hirsutism or cardiac disorders are other possible side effects of diazoxide treatment.^25^, ^26^ Somatostatin analogues are used as the second‐line treatment for diazoxide‐unresponsive cases of congenital HI. Lanreotide is a somatostatin long‐acting synthetic analogue that is used in the treatment of neuroendocrine tumors as well as in cases of diazoxide‐resistant or diazoxide‐unresponsive HI as it is an efficient and well‐tolerated alternative to achieve glycemic control.^27^ While being treated with diazoxide, our patient developed a digestive intolerance with symptoms such as vomiting and food aversion, which justified switching from diazoxide to lanreotide. It is important to note that digestive symptoms resulting from its inhibitory effect on the digestive system may be experienced by patients treated with lanreotide.^28^

Lanreotide binds to sst_2_ and sst_5_ receptors of the pancreas and inhibits the release of insulin.^29^ Its long‐acting form has a half‐life of 25.5 days^29^ and requires to be administered via an intramuscular or a deep subcutaneous injection every 4–6 weeks,^30^ which is a significant asset of the quality of life of the patients who beneficiate from it and their adherence to their treatments. The use of lanreotide has been proven to be efficient on the mean glycemia of patients with severe neonatal hyperinsulinism. However, no long‐term review on its efficacy is available.^31^

Overall, this case study reports the case of an X‐linked KS initially revealed by an HI. A syndromic HI should therefore be considered in a patient with persistent symptoms of hypoglycemia especially if associated with feeding difficulties and dysmorphic features. Diazoxide can be initiated first and somatostatin analogues such as lanreotide are good substitutes in cases of intolerance or unresponsiveness to diazoxide.

## AUTHOR CONTRIBUTIONS

**Saloua Ait Souabni:** Conceptualization; visualization; writing – original draft; writing – review and editing. **Antoine Harvengt:** Conceptualization; visualization; writing – original draft; writing – review and editing. **Camille Legat:** Conceptualization; visualization; writing – original draft; writing – review and editing. **Philippe A. Lysy:** Conceptualization; supervision; validation; visualization; writing – review and editing.

## FUNDING INFORMATION

This research was supported by clinical research funds from the Fondation Saint‐Luc and the Fonds de la Recherche Scientifique (FNRS).

## CONFLICT OF INTEREST STATEMENT

The authors declare no conflict of interest.

## CONSENT

Written informed consent from the patient's guardian/parent has been obtained to publish this report in accordance with the journal's patient consent policy.

## Data Availability

Available from corresponding author request.
